# Evaluation of cumulative prognostic scores based on the systemic inflammatory response in patients with inoperable non-small-cell lung cancer

**DOI:** 10.1038/sj.bjc.6601242

**Published:** 2003-09-09

**Authors:** L M Forrest, D C McMillan, C S McArdle, W J Angerson, D J Dunlop

**Affiliations:** 1 University Department of Surgery, Royal Infirmary, Glasgow G31 2ER, UK; 2 Department of Medical Oncology, Royal Infirmary, Glasgow G31 2ER, UK

**Keywords:** stage, performance status, albumin, C-reactive protein, non-small-cell lung cancer, cumulative prognostic score

## Abstract

A score based on the combination of the systemic inflammatory response and albumin hazards ratio (HR) 1.70, 95% CI 1.23 – 2.35, *P*=0.001) was comparable in prognostic value to that based on stage and performance status (HR 1.48, 95% CI 1.12 – 1.95, *P*=0.006) in patients with inoperable non-small-cell lung cancer. The former is simple to measure and well standardised.

Non-small-cell lung cancer is the most common cause of cancer death in North America and Western Europe. Most patients present with advanced inoperable disease and consequently the prognosis of these patients is extremely poor. Conventionally, the selection of patients for active or supportive treatment has been based on clinicopathological criteria, including age, stage, performance status, weight-loss or hypoalbuminaemia (Numico *et al*, 2001). However, the assessment of both performance status and the presence of weight loss may be subjective. For example, significant differences in the assessment of performance status have been reported between oncologists, nurses and patients, oncologists being the most optimistic in their assessment and patients the least (Ando *et al*, 2001).

Recent studies have shown that the presence and the magnitude of a systemic inflammatory response, as evidenced by increased circulating concentrations of C-reactive protein, is a prognostic factor independent of age, stage, performance status, weight-loss and hypoalbuminaemia in patients with advanced cancer including non-small-cell lung cancer (NSCLC) (Martin *et al*, 1999; O'Gorman *et al*, 2000; Mahmoud and Rivera, 2002; Scott *et al*, 2002).

The aim of the present study was to assess the value of combining C-reactive protein and recognised prognostic factors as stage, performance status and hypoalbuminaemia to form new prognostic scores for patients with inoperable NSCLC.

## MATERIALS AND METHODS

### Study design

Patients presenting with inoperable NSCLC (stages III and IV) to a single multidisciplinary oncology clinic in Glasgow Royal Infirmary between January 1997 and October 2002 were included in the study. Information was abstracted from the case notes by a specially trained research nurse (LMF). Data for 1997–1999 were collected retrospectively and that for 2000–2002 prospectively. All patients had cytologically or histologically confirmed disease and were staged on the basis of clinical findings, chest X-ray, and where appropriate, bronchoscopy, liver ultrasound, isotope bone scan and computerised tomography of the thorax, according to the American Thoracic Society TNM classification (Mountain, 1991).

Clinical stage, tumour type and performance status (Eastern Cooperative Oncology Group, ECOG) were recorded at the time of diagnosis. A blood sample was also obtained for measurement of haemoglobin, white cell count, albumin and C-reactive protein concentrations. Patients were considered to have undergone active treatment if they received chemotherapy (mainly cisplatin based) and/or radical radiotherapy. Patients receiving palliative radiotherapy and/or palliative care (symptom control) were considered to have had supportive treatment.

The study was approved by the Research Ethics Committee of Glasgow Royal Infirmary.

### Methods

#### Blood parameters

Routine laboratory measurements of haemoglobin, white cell count, albumin and C-reactive protein concentration were carried out. The coefficient of variation for these methods, over the range of measurement, was less than 5% as established by routine quality control procedures.

### Statistics

Data are presented as median and range. Grouping of the variables age, tumour type, performance status (ECOG), haemoglobin, white cell count and albumin was carried out using standard thresholds (Paesmans *et al*, 1997; Herndon *et al*, 1999). C-reactive protein concentrations were also grouped (⩽10/>10 mg l^−l^) as previously described (O'Gorman *et al*, 2000). Prognostic scores were constructed by assigning one point for each of the following criteria: stage IV, ECOG 2–4, albumin <35 g l^−l^ and C-reactive protein >10 mg l^−l^. Cumulative scores were obtained by combining C-reactive protein with each of the other variables.

Univariate survival analysis was performed using the Kaplan–Meier method. Multivariate survival analysis and calculation of hazard ratios (HR) were performed using the Cox regression analysis with prognostic scores as covariates. Deaths up to 31 January 2003 were included in the analysis. Analysis was performed using SPSS software (SPSS Inc., Chicago, IL, USA).

## RESULTS

The characteristics of patients with inoperable NSCLC (*n*=161) are shown in Table 1

**Table 1:** Clinical characteristics and survival in patients with inoperable NSCLC: univariate survival analysis

	**Patients 161 (100%)**	**Survival (months) Median (95% CI)**	***P* value**
Age			
<60 years	37 (23)	10.3 (8.2–12.4)	
⩾60 years	124 (77)	8.2 (6.1–10.3)	0.593
Sex			
Male	105 (65)	9.1 (6.6–11.6)	
Female	56 (35)	8.9 (5.9–11.8)	0.810
Stage			
III	57 (35)	12.0 (8.1–15.9)	
IV	104 (65)	6.9 (4.0–9.9)	0.015
Type			
Squamous	64 (40)	9.7 (5.9–13.5)	
Adenocarcinoma	53 (33)	9.0 (2.6–15.3)	
Other	44 (27)	3.9 (0.2–7.9)	0.003
Performance status (ECOG)			
0–1	91 (57)	12.0 (8.7–15.4)	
2–4	70 (43)	4.2 (1.6–6.7)	<0.001
Haemoglobin			
⩾12 g l^−l^	101 (63)	9.1 (6.3–11.9)	
<12 g l^−l^	60 (37)	8.9 (6.4–11.3)	0.187
White cell count			
⩽10 × 10^9^ l^−l^	82 (51)	12.4 (7.9–16.9)	
>10 × 10^9^ l^−1^	79 (49)	6.5 (2.9–10.0)	0.003
Albumin			
⩾35 g l^−l^	126 (78)	10.1 (7.4–12.8)	
<35 g l^−l^	35 (22)	3.7 (2.3–5.1)	<0.001
C-reactive protein			
⩽10 mg l^−l^	29 (18)	16.4 (11.0–21.7)	
>10 mg l^−l^	132 (82)	7.1 (4.9–9.3)	0.003
Treatment			
Active	63 (39)	13.3 (10.4–16.2)	
Palliative	98 (61)	4.7 (2.0–7.5)	<0.001

. The majority were male, over the age of 60 years, had stage IV disease and had good performance status. Approximately, 20% had hypoalbuminaemia and 80% had an elevated C-reactive protein concentration. One-third of patients received active treatment (64% received cisplatin-based chemotherapy); the remaining patients received supportive treatment.

In all, 118 (73%) of patients died during the follow-up period. On univariate survival analysis, stage (*P*<0.05), tumour type (*P*<0.01), performance status (*P*<0.001), white cell count (*P*<0.01), albumin (*P*<0.001) and C-reactive protein (*P*<0.01) were significant predictors of survival. On multivariate survival analysis all these variables, with the exception of white cell count, remained independent significant predictors of survival. There was a significant correlation between C-reactive protein and albumin concentrations (*r*_s_=−0.443, *P*<0.001).

On univariate analysis, the relationship between the prognostic scores based on the combinations of C-reactive protein with stage, performance status and albumin, respectively, and the median survival is shown in Table 2

**Table 2:** Cumulative prognostic scores and survival in patients with inoperable NSCLC (*n*=161): univariate survival analysis

**CRP/stage**	**(*n*)**	**CRP**	**Stage**	**Cumulative scores**	**Survival (months)**	**Hazard ratio (95%CI)**
	14	⩽10 mg l^−l^	III	0	18.2 (14.5–21.9)	
	15		IV	1	10.4 (7.9–12.8)	
	43	>10 mg l^−l^	III	1	8.9 (5.5–12.3)	1.73 (1.23–2.33)
	89		IV	2	6.1 (3.4–8.7)	*P*<0.001
CRP/ECOG		CRP	ECOG			
	23	⩽10 mg l^−l^	0–1	0	17.9 (15.5–20.3)	
	6		2–4	1		
	68	>10 mg l^−l^	0–1	1	9.0 (4.3–13.6)	1.79 (1.37–2.35)
	64		2–4	2	4.2 (2.2–6.2)	*P*<0.001
CRP/albumin		CRP	Albumin			
	27	⩽10 mg l^−l^	⩾35 g l^−1^	0	17.0 (11.4–22.6)	
	2		<35 g l^−1^	1		
	99	>10 mg l^−l^	⩾35 g l^−1^	1	8.9 (6.3–11.4)	2.00 (1.47–2.70)
	33		<35 g l^−1^	2	3.9 (0.8–7.1)	*P*<0.001
Stage/ECOG		Stage	ECOG			
	40	III	0–1	0	16.1 (9.5–22.6)	
	17		2–4	1	9.7 (3.5–15.8)	
	51	IV	0–1	1	10.4 (5.8–14.9)	1.73 (1.33–2.24)
	53		2–4	2	3.6 (1.7–5.6)	*P*<0.001

CRP=C-reactive protein; median survival (95% CI).

. All these prognostic scores had HR in the range 1.7–2.0 which was comparable with that for the score based on the combination of stage and performance status.

On multivariate analysis, when the three scores based on the combinations of the systemic inflammatory response and stage, performance status and albumin were compared with the combination of stage and performance status, only the score based on the combination of the systemic inflammatory response and albumin (HR 1.70, 95% CI 1.23–2.35, *P*=0.001) and the score based on stage and performance status (HR 1.48, 95% CI 1.12–1.95, *P*=0.006) retained independent significance.

## DISCUSSION

In the present study, both the established indicators of the presence of a systemic inflammatory response, white cell count and C-reactive protein, were predictive of survival in patients with inoperable NSCLC. This is consistent with previous studies, which have shown that a raised white cell count (Paesmans *et al*, 1997) and C-reactive protein concentrations (Scott *et al*, 2002), have prognostic value, independent of stage, in patients with inoperable NSCLC.

When C-reactive protein concentrations were combined with stage, performance status and albumin to form new prognostic scores, these combined scores improved the prediction of survival based on stage, performance status or albumin alone. Indeed, in each of the cumulative scores, the presence of an elevated C-reactive protein was associated with a halving of survival. Furthermore, when these three combinations were compared to the conventional combination of stage and performance status, the combination of the systemic inflammatory response and albumin and the conventional clinical combination stage and performance status were found to have comparable prognostic value. To our knowledge, this is the first study to present a cumulative prognostic score based on C-reactive protein and albumin in patients with advanced NSCLC.

In the present study, there was a significant difference in survival between those receiving active treatment and those receiving palliative care. However, this is not surprising since those selected for active treatment tend to be the fitter patients. Indeed, in the present study, 50% of patients with good performance status (ECOG 1–2) and 14% of those with poor performance status (ECOG 3–4) received active treatment. Treatment was not included in the prognostic model since the selection of patients for active treatment is based primarily on performance status.

These results are consistent with the evidence that chronic activation of the systemic inflammatory response is detrimental to the outcome of patients with NSCLC, being associated with an increase in weight loss (Staal van den Brekel *et al*, 1995; Scott *et al*, 1996) and fatigue (Scott *et al*, 2003), loss of lean tissue (McMillan *et al*, 1998; Simons *et al*, 1999), decreased performance status and survival (Martin *et al*, 1999; Scott *et al*, 2002).

The mechanism by which a systemic inflammatory response is evoked in patients with NSCLC is not clear. One possibility is that since many patients have coexisting pulmonary infection, the resultant increase in white blood cells would lead to increased proinflammatory cytokine release and a subsequent increase in circulating C-reactive protein concentrations. However, in this study, although the white cell count was significantly correlated with C-reactive protein concentrations, the magnitude of the relationship was small (*r*^2^ less than 15%). This would suggest that infection was not the main stimulus to the increased C-reactive protein concentrations.

An alternative explanation would be that proinflammatory cytokines are produced by the tumour. Indeed, there is evidence that proinflammatory cytokines are produced locally by tumours in patients with NSCLC (Arias-Diaz *et al*, 1994). In particular, interleukin-6 is recognised as a primary mediator of increased C-reactive protein concentrations in these patients (Yanagawa *et al*, 1995; Scott *et al*, 1996). However, whether interleukin-6 is produced directly by lung cancer cells remains unclear.

In conclusion, the results of the present study suggest that a cumulative score based on C-reactive protein and albumin may have prognostic value similar to that provided by more conventional measures. However, the cumulative score based on C-reactive protein and albumin has the advantage that of being simple to measure, routinely available and well standardised. Further large-scale prospective studies are required to confirm these findings and establish its value as a guide to the selection of patients for treatment.
